# Empirical evidence on structural racism as a driver of racial inequities in COVID-19 mortality

**DOI:** 10.3389/fpubh.2022.1007053

**Published:** 2022-11-22

**Authors:** Tyson H. Brown, Christina Kamis, Patricia Homan

**Affiliations:** ^1^Department of Sociology, Duke University, Durham, NC, United States; ^2^Center for Demography of Health and Aging, University of Wisconsin-Madison, Madison, WI, United States; ^3^Department of Sociology, Florida State University, Tallahassee, FL, United States

**Keywords:** structural racism, COVID-19 mortality, geographic inequality, racial inequality, health and mortality, measurement

## Abstract

**Objective:**

This study contributes to the literature by empirically testing the extent to which place-based structural racism is a driver of state-level racial inequalities in COVID-19 mortality using theoretically-informed, innovative approaches.

**Methods:**

CDC data are used to measure cumulative COVID-19 death rates between January 2020 and August 2022. The outcome measure is a state-level Black-White (B/W) ratio of age-adjusted death rates. We use state-level 2019 administrative data on previously validated indicators of structural racism spanning educational, economic, political, criminal-legal and housing to identify a novel, multi-sectoral latent measure of structural racism (CFI = 0.982, TLI = 0.968, and RMSEA = 0.044). We map B/W inequalities in COVID-19 mortality as well as the latent measure of structural racism in order to understand their geographic distribution across U.S. states. Finally, we use regression analyses to estimate the extent to which structural racism contributes to Black-White inequalities in COVID-19 mortality, net of potential confounders.

**Results:**

Results reveal substantial state-level variation in the B/W ratio of COVID-19 death rates and structural racism. Notably, regression estimates indicate that the relationship between the structural racism and B/W inequality in COVID-19 mortality is positive and statistically significant (*p* < 0.001), both in the bivariate model (adjusted R^2^ = 0.37) and net of the covariates (adjusted R^2^ = 0.54). For example, whereas states with a structural racism value 2 standard deviation *below* the mean have a B/W ratio of approximately 1.12, states with a structural racism value 2 standard deviation *above* the mean have a ratio of just above 2.0.

**Discussion:**

Findings suggest that efficacious health equity solutions will require bold policies that dismantle structural racism across numerous societal domains.

## Introduction

More than one million Americans have died from COVID-19. Notably, the impact of the pandemic has been unequally distributed across the color line. Racial inequities in COVID-19 mortality are well documented, with people racialized as Black experiencing much higher mortality rates than their White counterparts (1). Indeed, as of October 2022, the cumulative age-adjusted mortality rate for Blacks is 63% higher than it is for Whites (2). Consequently, Black Americans have experienced especially high levels of pandemic-related excess death (3, 4). The well-established disproportionate impact of the pandemic among Black the community has led to a great deal of discussion about the causes of these inequities.

Much of the scientific literature on Black-White COVID-19 mortality inequities has focused on the impact of *proximal causes*, such as racial inequalities in underlying health conditions, health care, and socioeconomic resources. A growing body of research, however, points to the role of upstream “causes of the causes” (5–7) undergirding the unequal toll of the pandemic along racial lines. In particular, numerous scholars have hypothesized that racial inequalities in COVID-19 mortality are driven by structural racism–i.e., a multi-sectoral, interrelated, system of racial oppression and exclusion from power, resources, opportunities, and well-being (8–11). This conceptualization aligns with accumulating evidence that discriminatory environments undermine the health of minoritized populations and contribute to racialized health outcomes (12–17). Theory highlights how structural racism indirectly harms the health of Black people because it leads to unequal access to salubrious resources and exposure to health risks (11, 18, 19). In the context of the COVID-19 pandemic, structural racism is thought to be an upstream cause of the downstream proximal causes (e.g., racial inequalities in underlying health conditions, economic and social deprivation, toxic living and working conditions, political exclusion, exposure to stressors, constrained autonomy and freedom, and inadequate health care) of Black-White inequities in COVID-19 mortality (7, 8, 20–22). Although a plethora of conceptual essays have hypothesized that structural racism is a driver of Black-White inequality in COVID-19 mortality (20, 23, 24), very few empirical studies have tested this proposition.

Robust empirical evidence of a relationship between areal structural racism and racial inequality in COVID-19 mortality would require at least three conditions:

1) geographic variation in racial inequalities in COVID-19 mortality.
2) geographic variation in structural racism.
3) a statistically significant relationship between structural racism and racial inequalities in COVID-19 mortality, net of likely confounders.

Below we summarize the evidence base for these three conditions, with a focus on limitations in prior research and how this study uses innovative approaches to improve our understanding of the extent to which structural racism is a driver of Black-White inequities in rates of COVID-19 mortality.

With respect to the first condition, prior research suggests that there is substantial variation in racial inequalities in COVID-19 mortality at both the county and state levels (21, 25–30). U.S. states are a particularly important geographic unit of analysis because, as Siegel and colleagues (2022) note, “*Understanding racial disparities at the state level is imperative because states have the primary responsibility for implementing policies related to the prevention, control, and response to COVID-19 and therefore are directly responsible for the emergence of, and amelioration of, racial disparities related to COVID-19*.” (30). Only a handful of quantitative studies have examined state-level variation in Black-White inequalities in COVID-19 mortality rates, and they are limited in several respects. For example, studies have often used crude death rates rather than rates that are age-adjusted (25, 29). Relying on crude death rates is problematic given the greater COVID-19 mortality risk among older adults in tandem with state differences in age distributions, as well as the younger population age profiles among Black Americans relative to their White counterparts. The few studies that have adjusted for age have often used indirect age standardization (24, 25), which is an inferior approach relative to direct age standardization because estimates based on indirect standardization are imprecise and are often not comparable across states (2). We are aware of only one published study on the topic that uses direct age standardization; findings show that not adjusting for age leads to severe underestimation of Black-White inequalities in COVID-19 mortality (30).

There is also growing evidence of state-level variation in structural racism. In fact, several studies have shown that indicators of structural racism—operationalized as Black-White inequities in societal domains such as housing, education, economics, politics, and the criminal-legal system—vary considerably across states, with levels of structural racism being particularly high in the Midwest and Northeast (31–34). These findings are consistent with the view that states are racialized institutional actors that shape the discriminatory, inequitable distribution of a plethora of social determinants of health along racial lines (35).

Regarding the third condition, a recent empirical study by Siegel and colleagues (2022) is the only one we are aware of that explored the association between state-level structural racism and Black-White inequities in COVID-19 mortality. Consistent with theory and hypotheses from a number of conceptual commentaries (7, 8), findings indicated that higher levels of structural racism—across multiple domains of society—were predictive of larger Black-White inequities (30). This was a very insightful contribution to the literature, yet the study had several limitations and there remain important gaps in our understanding of the extent to which structural racism is a driver of racial inequities in COVID-19 mortality. First, the Siegel et al. study includes information on deaths due to COVID-19 only through November of 2020—and thus does not capture the vast majority of deaths attributed to COVID-19 throughout the pandemic as it has continued to the present. Second, the study by Siegel and colleagues relies on bivariate associations that do not account for potential confounders of the relationship between structural racism and mortality due to COVID-19 (30). Third, the study uses a summative index of structural racism across societal domains rather than a latent variable approach, which has a number advantages for measuring structural racism (described below).

We aim to extend prior research and address these gaps in the literature by using a theoretically-informed, innovative approach to measuring state-level structural racism and its impact on racial inequalities in COVID-19 mortality. Specifically, we use up-to-date data on (directly) age-standardized COVID-19 deaths (through August 20, 2022), adjust for potential confounders, and develop a novel, multi-sectoral latent measure of structural racism. This latent variable approach has several advantages including 1) capturing the multifaceted, interconnected and systemic nature of the complex and often hidden phenomena of structural racism, 2) allowing for variance in factor loadings (rather than assuming monolithic weights for each of the observed indicators), 3) permitting covariances specified between observed indicator variables, and 4) minimizing measurement error (14, 35–37). Collectively, findings suggest that these approaches have considerable utility for population health research, and that state-level structural racism is a driver of place-based Black-White inequalities in COVID-19 mortality. This is consistent with a growing literature pointing to population health as a mirror reflecting societal arrangements.

## Methods

### Age-adjusted mortality rates

CDC WONDER data are used to measure racial inequality in cumulative COVID-19 death rates between January 1st 2020 and August 20th 2022. The outcome measure is a state-level (Non-Hispanic) Black-White (B/W) ratio of age-adjusted death rates (AADR), which are calculated using the direct method^1^. CDC WONDER calculates age-adjusted death rates using direct standardization with the “2000 U.S. standard” as the standard population (for more information see CDC WONDER data documentation) (41). Age-adjusted death rates are preferable over crude death rates (CDR) because age is linked to COVID-19 mortality risk and because there are racial differences in the age profiles of the population. Consistent with other studies on state-level structural racism, this study excludes 13 states, producing a sample of 37 U.S. States (30, 31). The 13 states excluded have insufficient information on the state's Black population due to a low proportion of Black residents (<4.6%) and/or a low total population of Black residents (<50 k residents). The 37 states included in the study represent 99% of the U.S. Black population and 93% of the U.S. white population.

### State-level structural racism indicators and latent scale

Consistent with research noting that U.S. states are racialized institutional actors shaping population health, and that structural racism involves multiple, interconnected societal domains (9, 10, 32, 35, 42), we utilize state-level 2019 administrative data on seven indicators of structural racism spanning educational, economic, political, criminal-legal and housing sectors. The indicators include: W/B ratios of Bachelor's degree, B/W ratios of poverty, W/B ratios of homeownership, B/W ratios of unemployment, W/B ratios of voting rates (in 2016 election), B/W ratios of incarceration, and the dissimilarity index of racial residential segregation (calculated at the state-level). A majority of these measures are derived from the U.S. Census Bureau's Current Population Study (CPS), with the exception of the measures of state-level residential segregation (data from America's Health Ranking) and incarceration (data from Bureau of Justice Statistics). Additionally, total population values were gathered from the American Community Survey 1-year estimates and used in the calculation of incarceration rates. Importantly, these seven indicators have been developed and validated in prior research (30, 32, 34, 35).

We use these validated measures to identify a novel, multi-sectoral latent measure of structural racism. Utilizing a latent measure of structural racism aligns with race theories positing that structural racism is systemic and often unobserved. We use confirmatory factor analysis (CFA) to estimate a series of latent constructs with varying specifications. We first examine a model in which each structural racism dimension is loaded onto a single factor. We then allow for errors to be correlated for several dimensions in subsequent models, based on an assessment of the correlation matrix and driven by theoretical considerations. Fit was assessed using chi-square, BIC, RMSEA, CFI, and TLI.

The first model, which includes each structural racism indicator loaded onto a single factor with no correlated errors, had a moderate fit. The Chi-square was non-significant, but the RMSEA was over .05 and the CFI/TLI were both below .9. Permitting the error terms for the inequity in incarceration and inequity in unemployment to correlate improved fit, but the RMSEA was still over .05 and the TLI was still under .9. Adding an additional term that allowed for the error terms for the inequity in homeownership and inequity in voting to correlate had a much better fit, (CFI = 0.982, TLI = 0.968, and RMSEA = 0.044). Additional specifications were considered (such as allowing all errors for economic measures to be correlated), but they did not produce substantive changes in fit and had higher BIC values, therefore we proceed with the model that includes each structural racism indicator loaded onto a single factor with correlated errors between incarceration and employment inequities and between voting and homeownership inequities. See Supplementary Figure S1 for a diagram of our measurement model with factor loadings and correlated errors. We note that analyses using the latent variable produced by the base model without any correlated errors produced similar results to those presented here, despite it's relatively worse model fit^2^. In addition to the latent measure, we considered a composite index that standardized and summed each of the individual indicators of structural racism. However, the latent variable model provided a better fit and a higher adjusted R-squared, indicating that it explained 24% more variation in our outcome (see the Supplementary Table S1 for additional details). While using a latent structural racism variable is the best approach for this study, it is possible that alternative approaches to measuring structural racism would be appropriate in other cases. Ultimately, the measurement of structural racism should be informed by research questions, logic, spatial and temporal contexts, feasibility, and data availability and fit.

### Covariates

To minimize the risk of biased estimates, this study accounts for a range of potential confounders. Consistent with prior studies, regression estimates control for several state-level factors, including: population size (logged), percentage of the population that is NH Black, Gini coefficient, poverty rate, and region (31, 32, 34, 35).

### Analyses

We begin by mapping B/W inequalities in COVID-19 mortality as well as the latent measure of structural racism in order to understand their spatial distribution across U.S. states. Next, we link the latent structural racism measure to CDC COVID data, and use Ordinary Least Squared (OLS) regression analyses to estimate the relationship between structural racism and Black-White inequalities in COVID-19 mortality. Multivariable analyses adjust for the covariates described above.

## Results

**Figure 1:**
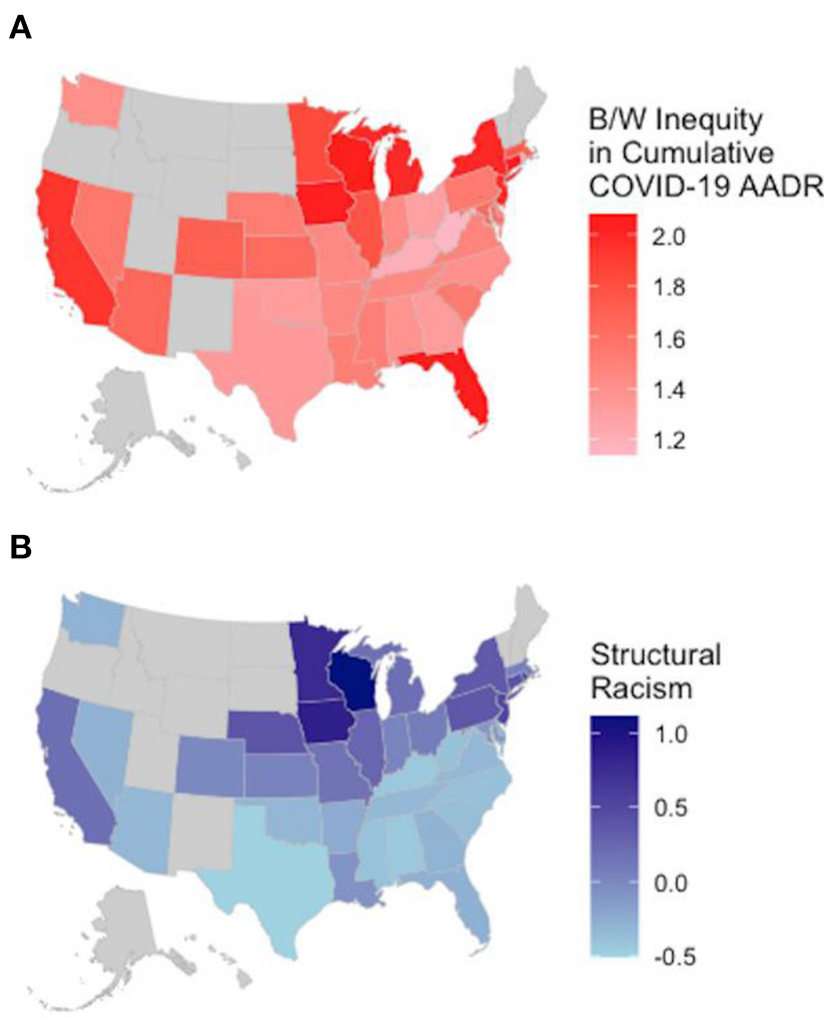
Geography of Black–White inequalities in cumulative COVID−19 mortality **(A)** and structural racism **(B)**, across U.S. States. **(A)** Shows B/W inequities in cumulative age–adjusted COVID−19 mortality (January 2020 to August 2022). **(B)** Shows a latent scale of state–level structural racism in 2019 spanning educational, economic, political, criminal–legal and housing domains.

Table 1 provides descriptive statistics and information about the sources for each of the study variables. The average ratio of B/W COVID-19 mortality suggests that for U.S. states, there are more Black deaths than white deaths. There are also B/W inequities across all measures of structural racism that indicate a larger burden on Black populations. Figure 1 includes maps showing substantial state-level variation in the B/W ratio of age-adjusted COVID-19 death rates (Figure 1A) and structural racism (Figure 1B), respectively. B/W ratios of COVID-19 mortality range from 1.14–2.08, with the greatest inequalities in upper midwestern and northeastern states. This means that in all states, Black COVID-19 death rates were substantially higher than white death rates. Similarly, mapping the spatial distribution of structural racism reveals that, despite its ubiquity, it tends be especially elevated in midwestern and northeastern states. This is consistent with an emerging body of literature on the spatial distribution of structural racism across U.S. states (30, 31, 35, 43–45). Although the historical and modern roots of state differences in structural racism are not fully understood, scholars have posited that elevated levels of contemporary structural racism—manifest in discriminatory institutional contexts—in the Midwestern and Northeastern states stem, in part, from institutionalized policies and practices of social control through racialized exclusion and subordination such as resource hoarding, redlining, racial covenants and discriminatory policing. These white supremacy tactics were increasingly deployed in response to the Great Migration because Northern Whites perceived the growing Black population as a threat (33, 35, 43, 44, 46).

**Table 1:** Descriptive statistics of U.S. States (*N* = 37).

	**Mean (or %)**	**SD**	**Range**	**Source**
Cumulative age-adjusted death rates (AADR; 1/2020-8/20/2022)				
B/W AADR	1.59	0.27	[1.14, 2.08]	CDC Wonder
State-level structural racism indicators (2019)				
B/W incarceration rates	5.74	2.58	[2.51, 12.26]	BJS; ACS 1-year estimate; Author's Calculations
W/B college degree completion	1.74	0.38	[1.01, 2.96]	CPS ASEC; Author's Calculations
B/W unemployment rates	2.36	0.87	[0.77, 4.77]	CPS ASEC; Author's Calculations
B/W poverty rates	2.70	0.87	[1.12, 4.97]	CPS ASEC; Author's Calculations
W/B homeownership rates	1.93	0.53	[1.35, 3.62]	CPS ASEC; Author's Calculations
W/B voting rates	1.14	0.24	[0.86, 1.90]	CPS Voting Supplement; Author's Calculations
B/W segregation	57.57	8.28	[42.00, 72.00]	America's Health Ranking
				
**State-level structural racism (2019)**				
Latent structural racism	0.00	0.41	[-0.51, 1.11]	
				
**Covariates**				
Logged total population (2019)	15.61	0.82	[13.79, 17.49]	ACS 1-year estimate
Percentage NHB (2019)	15.53%		[4.59%, 38.58%]	CPS ASEC
Gini (2019)	0.47	0.02	[0.44, 0.51]	ACS 1-year estimate
Percentage below the poverty line (2019)	11.75%		[7.40%, 19.57%]	CPS ASEC
Region				U.S. Census
Northeast	16.22%			
Midwest	27.03%			
South	43.24%			
West	13.51%			

**Figure 2:**
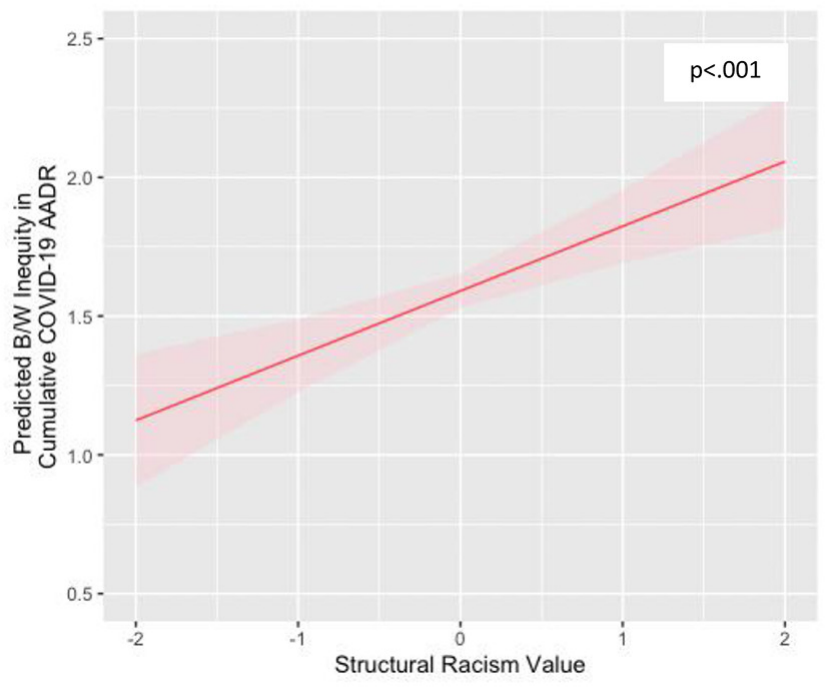
Relationship between state–level structural racism and Black-White inequities in COVID-19 mortality. Estimates account for potential confounders, including population size, percentage NH Black, Gini coefficient, poverty rate, and region.

Regression estimates in Table 2 indicate that the relationship between the structural racism and B/W inequality in COVID-19 mortality is statistically significant (*p* < 0.001), both in the bivariate model (Model 1; adjusted R^2^ = 0.37) and net of the covariates (Model 2; adjusted R^2^ = 0.54). Figure 2 graphically illustrates the predicted values of Black-White inequality in COVID-19 deaths as a function of structural racism using estimates from Table 2, Model 2 and holding all other covariates at their mean values. The figure shows that, higher levels of structural racism predict larger B/W ratios of COVID-19 death rates. For example, whereas states with a structural racism value 2 standard deviations *below* the mean have a B/W ratio of approximately 1.12 (for every one White death, there are 1.12 Black deaths), states with a structural racism value 2 standard deviations *above* the mean have a ratio of just above 2.0 (for every one white death, there are just above 2 Black deaths). States with the average structural racism value have a B/W ratio of 1.6.

**Table 2:** OLS regression predicting B/W inequities in cumulative COVID-19 Age-Adjusted Death Rates (AADR) by state-level structural racism (*N* = 37, U.S. States).

	**Model 1**	**Model 2**
	**Coef (SE)**	**Coef (SE)**
Latent structural racism	0.402^***^	0.563^***^
	(0.086)	(0.136)
		
Logged total population (2019)		0.046
		(0.046)
Percentage NHB (2019)		0.247
		(0.471)
Gini (2019)		8.710^*^
		(3.833)
Percentage below the poverty line (2019)		−3.713^*^
		(1.603)
Region		
South (ref.)		
Northeast		−0.334
		(0.174)
Midwest		−0.095
		(0.132)
West		0.086
		(0.122)
		
Constant	1.591^***^	−2.748
	(0.035)	(1.367)
BIC	−4.327	1.254
Adjusted R–squared	0.369	0.537

* p < 0.05;

^**^p < 0.01;

*** p < 0.001.

## Discussion

Racial inequality in mortality is an enduring hallmark of the U.S. population health landscape. For as long as U.S. mortality data have been collected, Black people have experienced higher rates of mortality than their white counterparts (4, 47). Mortality rates during the COVID-19 pandemic are no exception. While the COVID-19 pandemic has led to significant excess deaths across all racial groups in the U.S., its deadly effects have not been spread over a level playing field (4, 8, 30). Numerous scholars have hypothesized that structural racism is the root cause of the disproportionately high rates of COVID-19 mortality among Black people (7, 8, 48).

The vast majority of studies on the role of structural racism in driving racial inequalities in COVID-19 mortality rates have been conceptual, leading to a dearth of empirical evidence on the topic. This study contributes to the literature by empirically testing the extent to which place-based structural racism undergirds state-level racial inequities in COVID-19 mortality using innovative approaches. Our theoretically-informed latent measure of structural racism allowed us to better capture the multifaceted, interconnected and systemic nature of racism, providing a more robust picture of its health consequences. In addition to an innovative approach to measuring structural racism, this study extends prior research by analyzing up-to-date mortality data (through June of 2022) and adjusting for potential confounding factors. We found that while all states had higher rates of Black COVID-19 mortality than white COVID-19 mortality, higher levels of structural racism were associated with larger Black-White inequalities in COVID-19 mortality. In other words, the more racism imbedded in state-level institutions the worse Black residents fared, relative to their white counterparts. Taken together, our findings provide empirical support for research theorizing a connection between racism and COVID-19 outcomes, and add to a growing literature documenting harmful health consequences of structural racism (22, 37, 49, 50).

Evidence that structural racism is a driver of racial inequalities in COVID-19 mortality is critical for shifting the focus from untenable cultural deficit explanations—which blame the victims of White supremacy—toward the upstream root causes of the mortality inequities. Examining how unequal exposure to health-damaging social contexts, in general, and discriminatory environments in particular, aligns with prominent conceptual frameworks (e.g., Fundamental Cause Theory; Ecosocial Theory; the WHO Structural Determinants of Health framework) (11, 18, 51), as well as an emerging body of empirical research on the topic (21, 30). It is becoming more and more clear that Black-White inequities in population health reflect racialized societal arrangements across many sectors of society, including educational, economic, housing, political, and criminal-legal domains (9, 10, 42, 52).

As political, legal, administrative units, U.S. states play a key role in shaping the unequal distribution of social determinants of health (53–55). Moreover, findings from this study—in tandem with a nascent but growing body of research (30–32, 34, 35) —point to the importance of conceptualizing states as *racializing* institutional actors that shape population health. While structural racism is embedded in all states, results from this study reveal that states vary in their degrees of structurally racist contexts see also Siegel et al. (30). This is consistent with Bruch and colleagues' (57:163) contention that, “The state in which one resides has significant consequences for one's opportunities and life conditions and… for the structure of racial relations one must traverse” (56). Indeed, since the founding of the country states have been influential in sanctioning, exacerbating and alleviating racial oppression—from the historical roles they played with respect to policies on slavery, Jim Crow, and anti-miscegenation to their contemporary “race-neutral” policies that perpetuate racial domination such as voter disenfranchisement, gerrymandering, welfare state contraction and criminal sentencing laws (45, 57–59).

Our study has a number of limitations that point to fruitful avenues for future research. First, our study only contains state-level data. Although states are clearly a vital unit of analysis for understanding the mortality effects of structural racism, future research should seek to incorporate multilevel data to allow for the examination of individual-level exposures and outcomes, as well as structural racism at organizational, neighborhood, county, state and regional levels. Second, our data do not permit testing of the more proximal mechanisms connecting structural racism to COVID-19 deaths. To the extent that rich multilevel data become available, research should examine the theorized pathways through which racism is expected to increase risk of COVID-19 death, including: chronic health conditions, economic and social deprivation, toxic living and working conditions, political exclusion, inadequate health care, and psychosocial factors (e.g., social stressors, lack of autonomy, and stigma) (7, 11, 18, 30). Third, while we have employed a relatively comprehensive measure of structural racism across multiple institutional domains, it does not represent an exhaustive analysis of all the ways systemic racism shapes health. In addition to the institutional aspects of structural racism we examined, future research should also investigate the health effects of historical and contemporary discriminatory laws and policies, as well as anti-black cultural orientations and ideologies (60–62). Fourth, although this study is focused on understanding the dramatic Black-White inequities in COVID-19 deaths observed in the US, it also important for future research to examine race-specific COVID-19 death rates and whether there is evidence that elevated levels of structural racism are universally harmful. Studies examining other types of health outcomes have tended to find no effect of state-level structural racism among whites (31, 34, 35), but at least one has found evidence of a health benefit for whites (32). Finally, we focused on anti-Black structural racism because it has been a central and enduring feature of American society, but there is a need for future research to examine the impact of additional forms of structural racism on an array of racialized groups.

The COVID-19 Pandemic is shedding light on U.S. mortality inequities across the color line, leading to a growing understanding that structural racism is the root cause. Fundamental cause theory describes how societal forces (such as structural racism) shape the distribution of a multitude of health-relevant risks and resources and are therefore consistently linked to multiple disease outcomes through an array of mechanisms (18). Resources are flexible and can be leveraged to avoid disease even under changing circumstances, such as the COVID-19 pandemic. Thus, interventions to reduce racialized health inequities will be ineffective if they focus primarily on “proximal causes” of disease–which prove to be transient over time—rather than addressing structural racism as the more distal, fundamental cause. As new health threats emerge in the future—whether they are infectious diseases, environmental or climate related hazards, or even political-legal barriers to accessing necessary healthcare—we will continue to experience the same type of dramatic racial inequities we have seen during the COVID-19 pandemic unless we find ways to dismantle structural racism. Healthcare plays an important role in treating health problems and supporting population health, yet it is also critical to create social conditions that prevent (not just treat) health problems that disproportionately burden Black people in the US. Our study findings point to equity-promoting policies in social, economic, and political systems as necessary for creating conditions to achieve racial health equity. Research showing that Black-White inequalities in COVID-19 mortality (as well as other health outcomes) are a function of a multi-sectoral and reciprocal system of structural racism suggests that incremental policies that focus on a single domain are unlikely to substantially reduce racial inequalities (10, 18, 19, 30, 42, 50). Thus, efficacious health equity solutions will require bold policies that dismantle structural racism across numerous societal domains such as criminal justice reform, shoring up voting rights and eliminating felony disenfranchisement, implementing baby bonds to reduce the racial wealth gap and a federal jobs guarantee to close employment and earnings gaps, and reforming the public education finance system to promote racial equity in schools (15, 63, 64).

While the COVID-19 pandemic is an unprecedented global public health emergency, the racial inequities that have emerged in the United States are following well-known and predictable patterns. People racialized as Black continue to bear a disproportionate burden of disease and death. This represents an enormous amount of unnecessary and unequal human suffering that demands redress.

## Data availability statement

The datasets presented in this study can be found in online repositories. The names of the repository/repositories and accession number(s) can be found in the article/Supplementary material.

## Ethics statement

The studies involving human participants were reviewed and approved by Duke IRB. The patients/participants provided their written informed consent to participate in this study.

## Author contributions

TB identified the research question, developed the research plan, provided input on the analyses, and took the lead role in writing the manuscript. CK led on the data analysis and visualizations, and both CK and PH contributed to writing the manuscript. All authors contributed to the article and approved the submitted version.

## Funding

This research received support from grants P30 AG034424 (awarded to the Center for Population Health and Aging at Duke University by the National Institute on Aging), T32 AG00129 (awarded to the Center for Demography of Health and Aging at the University of Wisconsin-Madison by the National Institute on Aging), and 2R24AG045061-06 (awarded to the Network on Life Course Health Dynamics and Disparities in 21st Century America by the National Institute on Aging).

## Conflict of interest

The authors declare that the research was conducted in the absence of any commercial or financial relationships that could be construed as a potential conflict of interest.

## Publisher's note

All claims expressed in this article are solely those of the authors and do not necessarily represent those of their affiliated organizations, or those of the publisher, the editors and the reviewers. Any product that may be evaluated in this article, or claim that may be made by its manufacturer, is not guaranteed or endorsed by the publisher.

## Footnotes

^1^In assessing the quality of COVID, there is some evidence of unevenness in reporting COVID deaths across states (38, 39). One of the benefits of using a ratio measure of B:W COVID mortality (rather than overall or single race-specific) for our outcome is that even if reporting irregularities are systematic across states, the same “noise” may be present for both Black and White deaths within the state, in which case the validity of the measure of racial inequities in COVID would be unbiased. Furthermore, to the extent that there are racial differences in reporting, it would likely be under-reporting of Black deaths which would lead to conservative estimates of inequities (39, 40). Thus, we are unaware of any evidence to suggest that our findings of a relationship between structural racism and B-W COVID mortality inequities are biased due to data irregularities.

^2^We have assessed additional models that specified the relationship between structural racism and B/W disparities in cumulative COVID-19 AADR as quadratic and one that include structural racism as a categorical measure (quartiles of structural racism). The linear model (our current final model) resulted in a better fit for the data (lower BIC), providing support for a linear relationship.
