# Supplementary material for: Empirical evidence on structural racism as a driver of racial inequities in COVID-19 mortality
Source: Front Public Health. 2022 Nov 22;10:1007053. doi: 10.3389/fpubh.2022.1007053 (PMC9723349; doi:10.3389/fpubh.2022.1007053)
Supplement: Supplementary file 1 [file Data_Sheet_1.docx]

**Supplementary Material**

| **Supplementary Table S1. Fit Comparisons of Latent and Summative Index Measures of State-Level Structural Racism** | | |  |  |
| --- | --- | --- | --- | --- |
|  | Adjusted R-squared | BIC |  |  |
| Latent Measure of Structural Racism | 0.537 | 1.254 |  |  |
| Summative Index of Structural Racism | 0.434 | 8.666 |  |  |
| Models control for population size, percentage NH Black, Gini coefficient, poverty rate, and region. | | |  |  |

Table S1 shows the model fit statistics of regression analyses of Black-White inequities in COVID-19 mortality for models that used the latent measure of structural racism as well as a composite index that standardized and summed each of the individual indicators of structural racism. Results show that the latent variable provided a better fit (lower BIC values) and a higher adjusted R-squared, indicating that it explained 24% more variation in Black-White inequality in COVID-19 mortality. We also conducted ancillary analyses that revealed that models using the latent measure had lower BIC values and explained more variance than models that relied on individual indicators of structural racism. While a latent variable of structural racism is the preferred measurement approach for this study, alternative measurement approaches may be appropriate in other cases. Strategies for measuring structural racism should be informed by research questions, logic, spatial and temporal contexts, feasibility, and data availability and fit.

**Supplemental Figure S1. CFA Measurement Model of Structural Racism**


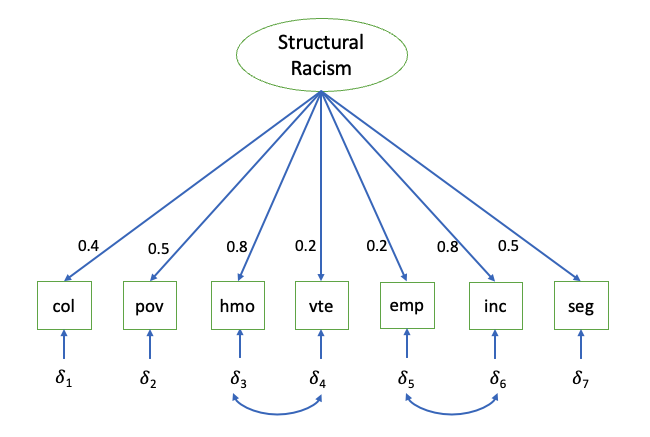


Note: The diagram of the CFA measurement model includes factor loadings and correlated errors among state-level indicators of structural racism as measured by racialized patterns in bachelor’s degrees (col), poverty (pov), home ownership (hmo), voting (vte), employment (emp), incarceration (inc) and segregation (seg). CFI=.982, TLI=.968, and RMSEA=.044. Additional details on the measurement model are included in the text.
